# The effect of platelet-rich fibrin (PRF) versus freeze-dried bone allograft (FDBA) used in alveolar ridge preservation on the peri-implant soft and hard tissues: a randomized clinical trial

**DOI:** 10.1186/s12903-024-04478-1

**Published:** 2024-06-14

**Authors:** Hassan Azangookhiavi, Sareh Habibzadeh, Houyar Zahmatkesh, Ehsan Mellati, Seyed Ali Mosaddad, Yalda Dadpour

**Affiliations:** 1 Department of Prosthodontics, School of Dentistry, International Campus, Tehran University of Medical Sciences, Tehran, Iran; 2 Dental Research Center, Dentistry Research Institute, Tehran University of Medical Sciences, Tehran, Iran; 3 Private Practice, Tehran, Iran; 4 University of Sydney, Sydney, Australia; 5 Private Practice, Sydney, Australia; 6 Department of Research Analytics, Saveetha Institute of Medical and Technical Sciences, Saveetha Dental College and Hospitals, Saveetha University, Chennai, India; 7 Department of Periodontics, International Campus, School of Dentistry, Tehran University of Medical Sciences, Tehran, Iran; 8 Department of Conservative Dentistry and Bucofacial Prosthesis, Faculty of Odontology, Complutense University of Madrid, Madrid, Spain

**Keywords:** Alveolar Ridge Augmentation, Bone substitutes, Alveolar Bone Grafting, Platelet-Rich Fibrin, Allografts

## Abstract

**Background:**

The effectiveness of alveolar ridge preservation on bone regeneration and tissue healing has been thoroughly documented in the literature. This study aimed to evaluate the peri-implant soft and hard tissue changes after alveolar ridge preservation using either platelet-rich fibrin (PRF) or freeze-dried bone allograft (FDBA) over a 12-month period following the prosthetic loading of implants.

**Methods:**

In this randomized clinical trial, 40 individuals were recruited for alveolar ridge preservation using (1) FDBA or (2) PRF in incisal/premolar areas. At two follow-up sessions (six- and 12-months post-implant insertion), radiographic imaging and clinical examinations assessed marginal bone loss and soft tissue factors, including gingival recession and bleeding on probing. The differences between study groups were analyzed using Generalized estimating Equations, the Binary logistic regression model, and Cochran’s Q test.

**Results:**

There was a statistically significant difference regarding gingival recession at both follow-up evaluations; values in the PRF group were considerably lower compared to the FDBA group (*p* < 0.05). The mean values for vertical marginal bone loss and bleeding on probing showed no significant differences between the two study groups (*p* > 0.05).

**Conclusions:**

Except for gingival recession, applying PRF yielded comparable clinical results to FDBA after one year of implant loading and could be recommended as a potential biomaterial for alveolar ridge preservation following tooth extractions.

**Clinical trial registration:**

The research protocol was registered in the Protocol Registration and Results System on 13/08/2021, available at https://clinicaltrials.gov/ (NCT05005377).

## Background

The loss of residual alveolar ridge volume could inevitably occur following tooth extraction [1] by losing dimension horizontally and vertically due to the complicated bone turnover process [2]. The resorption of bony ridge volume necessary for effective implant therapy complicates tooth replacement, adversely affecting the treatment plan and the outcome of implant-based therapies [3]. As dental implant success principally depends on the interactions of the implant with the adjacent hard and soft tissues [4–6], post-extraction sequels are required to be managed and maintained meticulously to obtain successful results and predictably reestablish function and aesthetics [7].

The peri-implant marginal bone is the most susceptible zone to resorption after implant placement. Early crestal bone loss often occurs during the first 12 months after implant placement and progressively continues at a lower rate [8]. Several factors are believed to be implicated in crestal bone loss, such as peri-implant soft tissue health [9], the reorganization of the periodontal biologic width, and the implant crest module [8]. Bone quality is another vital factor related to the rate of MBL around dental implants. A poor-quality grafted bone around implants, compared to a residual native one, can negatively affect the crestal bone due to the higher strain distribution at the crestal level. So ideally, the grafted bone should be just as or even more stiff than the adjacent native bone [10].

Several surgical techniques and different biomaterials are used to optimize the quantity and quality of the bone for implant-based treatments. One of these augmentation techniques is alveolar ridge preservation (ARP) [11, 12]. In this method, the extraction socket is filled with biomaterials to compensate for the physiologic remodeling of the bone [13]. This procedure makes implant operation easier and might eliminate the need for additional bone augmentation in the future when implant placement is delayed for the patient due to site-related reasons [1].

Two leading ends are involved in ARP; the first is to prevent ridge resorption from disrupting the ridge’s dimensional integrity. The second objective is to create vital bony tissue at the location of the extracted root to aid in placing implants and achieving adequate osseointegration [14]. In ARP, numerous biomaterials and procedures are used to accomplish these two objectives, including autogenous bone, bone substitutes such as allografts, xenografts, synthetic compounds, autologous blood products, and bioactive materials [1, 15].

Freeze-dried bone allograft (FDBA) is a commonly used biomaterial in ARP. Due to their osteoconductive qualities, simple application [16], ability to preserve the ridge’s dimensional stability, lack of need for a second surgery or donor sites, low cost, broad accessibility, and lower resorption rates than some other materials, FDBAs are becoming increasingly popular [1]. However, their usage has several drawbacks, such as infection transfer risk [17, 18]. Although rare, an increased chance of infection in sites with low vascularity has been reported, attributed to its slow revascularization by the creeping substitution process [19]. Allograft material’s increased shelf life and reduced immunogenicity can be achieved through freeze-drying but at the expense of osteoinductive potential, structural strength, and osseointegration [20].

Second-generation platelet substrate platelet-rich fibrin (PRF) is a dense scaffold of a fibrinous matrix polymerized in a four-molecule structure. It is non-toxic and non-immunogenic and is abundant in autogenous growth factors like TGF-β1, PDGF-AB, VEGF, platelet and leukocyte mediators like IL-1β, IL-4, and IL-6 [21], cytokines, and circulating stem cells [22]. PRF has been considered another promising material in filling bony defects, benefiting from stimulating osteoblastic differentiation and proliferation, which reinforces adjacent bone and accelerates bone regeneration by gradually releasing autologous growth factors [23–26]. It could enhance the early stages of bone regeneration by delivering a high concentration of growth factors more than their physiological level to the surgical site without the risk of infection transfer as allografts [27, 28]. PRF membranes can inhibit osteoclastogenesis from hematopoietic progenitors in bone marrow cultures and have favorable results in ARP [29].

Many studies indicated PRF superiority for alveolar ridge preservation regarding the bone fill percentage and marginal bone level stability [28, 30]. Several clinical studies have also confirmed the short-term benefits of PRF application in ARP after tooth extraction [11, 31, 32]. Still, limited reports investigate its efficacy after implant loading compared to other graft biomaterials and scaffolds. Considering the optimal effectiveness and potential value of FDBA and PRF in bone regeneration [33, 34], this study aimed to assess bone loss and soft tissue changes around implants following alveolar ridge preservation with these two biomaterials during the first year of loading. The null hypothesis was that the two groups would have no significant differences in marginal bone loss and soft tissue indices.

## Methods

This study was designed as a randomized clinical trial. The Research Ethics Committee of Tehran University of Medical Sciences authorized the present study (IR.TUMS.DENTISTRY.REC.1396.2610). In addition, the research protocol was registered in the Protocol Registration and Results System on 13/08/2021, available at https://clinicaltrials.gov/ (NCT05005377). The Declaration of Helsinki was also followed when conducting the research.

### Patient selection

The research included 40 subjects who needed single-tooth extractions and subsequent implant therapy. Patients referred to the Department of Periodontics at the Tehran University of Medical Sciences, who were required to extract a hopeless incisor/single-rooted premolar and receive a dental implant afterward, were considered for enrolling in the study. The causes of tooth extraction included inadequate crown-to-root ratio, extensive non-restorable caries, failed root canal treatment, and fractured roots. A single periodontist with more than five years of clinical experience (Y.D.) screened the subjects based on the inclusion criteria: having 18 years old or more, adequate systemic health status (ASA I and II), plaque index < 25%, and sufficient mesiodistal and interocclusal space. Patients with a history of systemic diseases, radiotherapy to head and neck, chemotherapy, conditions/medications that affect bone metabolism, smokers, patients with untreated periodontal diseases, teeth having periapical or periodontal lesions, teeth with buccal bone defects (dehiscence or fenestration), patients with thin gingival biotype, and keratinized gingiva < 4 mm around the teeth were excluded.

All patients recruited to be included in this study were first informed about the trial’s details and signed informed consent before enrollment. Patients could leave the research at any moment, and they ensured their information was confidential.

The sample size was calculated using GPower to be at least 15 participants in each group considering type one error of 0.05, power of 0.8, and effect size equal to 1.095 based on a previous study [32]. Therefore, considering the 20% dropout, the final sample size was 18 participants in each group.

Patients who met the requirements were randomly divided into a pair of groups with a 1:1 allocation ratio: Group 1 (*n* = 20): Alveolar ridge preservation using FDBA; Group 2 (*n* = 20): Alveolar ridge preservation using PRF.

### Surgical procedures

Patients were anesthetized with buccal and palatal infiltration anesthesia using an anesthetic solution containing lidocaine HCl 2% + epinephrine 1:100,000 (Persocaine-E®, Darou Pakhsh, Iran). Without flap elevation, a periotome elevator (Aesculap, USA) was used to extract the teeth with minimum trauma. The periotome was introduced around the root’s circumference to expand the socket’s bony walls. Finally, forceps were used gently to remove the teeth as atraumatically as possible. Following the atraumatic teeth extraction, the buccal and lingual bone plates were assessed under magnification (magnification loupe ×3.5) for bone defects to exclude sockets with bone dehiscence, fenestration, or fracture.

Random allocation software was used to produce a random sequence, and a randomized allocation table was generated through balanced block randomization. The research statistics partner created the randomized allocation list, and the practitioner distributed sequentially numbered sealed envelopes labeled “A” or “B” to patients before applying ARP with either FDBA (envelope A) or PRF (envelope B). Control and test sites were chosen randomly using the randomization list. The surgeon became aware of the group (control or test) immediately when performing the procedure; hence, blinding was not possible due to the detectability of surgery outcomes by both the patient and surgeon.

In the first group, the extraction sockets were filled using FDBA (150–1000 μm, CenoBone®; Tissue Regeneration Corp., Kish Island, Iran) without elevating the flap. A resorbable collagen sponge (Spongostan® Dental, Ferrosan, Denmark) was used to cover the socket [35, 36].

For the second group, 10 ml of the patient’s venous blood was obtained from the antecubital area. The sample was centrifuged in an IntraSpin machine (Intra-Lock International Inc., Boca Raton, FL, USA) for 12 min at 2700 rpm to obtain PRF, based on a procedure described by Dohan et al. [21]. One to three PRF clots were placed inside the extraction socket and enclosed with a PRF membrane.

A 4 − 0 monofilament nylon suture (Supalon, Supa, Iran) was applied to all sockets with a figure-of-eight design for securing the collagen sponge or the PRF membrane over the socket during the early healing phase. Each patient was surgically managed in one single session. Post-operation instructions were given verbally and in written form to all patients. Painkillers (ibuprofen, 400 mg, for two days, three times per day), antibiotics (amoxicillin, 500 mg, three times a day for five days), and mouthwash (chlorhexidine, 0.2%, twice daily for one week) were prescribed for all patients. The sutures were removed 14 days after the surgery.

Four months later, all patients received dental implants at the preserved sites. Following a full-thickness flap elevation, implant sites were prepared according to the protocol (25 Ncm, 800 rpm) proposed by the implant manufacturer (Zimmer Biomet, USA). The implants used in this study were Tapered Screw-Vent® bone-level implants (Zimmer Biomet, USA) with a threaded root-form macro design coated with an MTX® surface. The size of the implants was determined according to the existing bone dimensions (width and length) evaluated by preoperative examinations radiographically by cone-beam computed tomography (CBCT) images [37] and clinically by determining the available interdental space. A notable attempt was made to maintain > 1 mm of buccal bone during the insertion of the implants. Additionally, the implants were positioned about 3 mm below the apical margin of the eventual restoration and at least 1.5 mm away from the neighboring teeth. Implants were tightened with a torque wrench until fixtures had primary stability of at least 25 N centimeters (Ncm). All subjects needed no more augmentation procedures for implant insertion. All implants were left in place for three months to achieve proper osseointegration.

In the third stage of surgery, a crestal incision was made on the sites of placed implants in such a way as to maintain at least 2 millimeters of keratinized gingiva at the implants’ buccal surface. Following the elevation of a full-thickness flap, a gingival former (Zimmer Biomet, USA) that matched the attachment size was positioned.

One experienced periodontist (Y.D.) performed all surgical procedures using similar and standardized protocols to remove the effect of operative variables.

### Prosthetic protocol

Soft tissue healing around the gingival former was allowed for four weeks, and then a fixture-level impression was made using a closed-tray technique with additional silicone material (Panasil, Kettenbach GmbH & Co. KG, Eschenburg) as per its manufacturer’s instructions.

Straight abutments (Hex-Lock Contour Straight Abutment, Zimmer Biomet, USA) with a gingival height of 0.5 mm were selected for all restorations. In case of different gingival heights surrounding an implant, the highest level was selected, and the other abutment surfaces were prepared using a tapered diamond bur with a final finish line subgingivally positioned 0.5 mm.

After preparing the abutments, a resin pattern (GC Corporation, USA) was used to wax up a metal framework for a cement-retained metal-ceramic crown. A cobalt-chromium alloy (Wirobond® C, Bego, Germany) was used to cast the framework. Throughout the second session, the abutments and metallic frameworks were clinically evaluated. In the third session, intraoral adjustments of the fused porcelain (VITA VM ceramic, VITA Zahnfabrik, Germany) involving facial/lingual contours, interproximal contacts, occlusion, and esthetic appearance of the restoration were clinically performed. Afterward, the restorations were glazed per the manufacturer’s recommendations (VITA Akzent Plus Glaze LT; Vita Zahnfabrik, Germany).

In the final prosthetic session, abutments were tightened to a torque of 25 Ncm using a new screw (Zimmer Biomet, USA). A Teflon band was used to seal access holes, and temporary cement (TempBond, Kerr) was utilized to cement the glazed restorations. The extra cement was then removed completely. The thorough elimination of excess cement and seating of the prostheses were radiographically checked (baseline radiograph). All these prosthetic procedures were conducted by a prosthodontist (S.H.) with over five years of clinical experience and blinded to the type of biomaterial employed in the alveolar ridge preservation stage.

### Radiographic and clinical evaluations

**Fig. 1:**
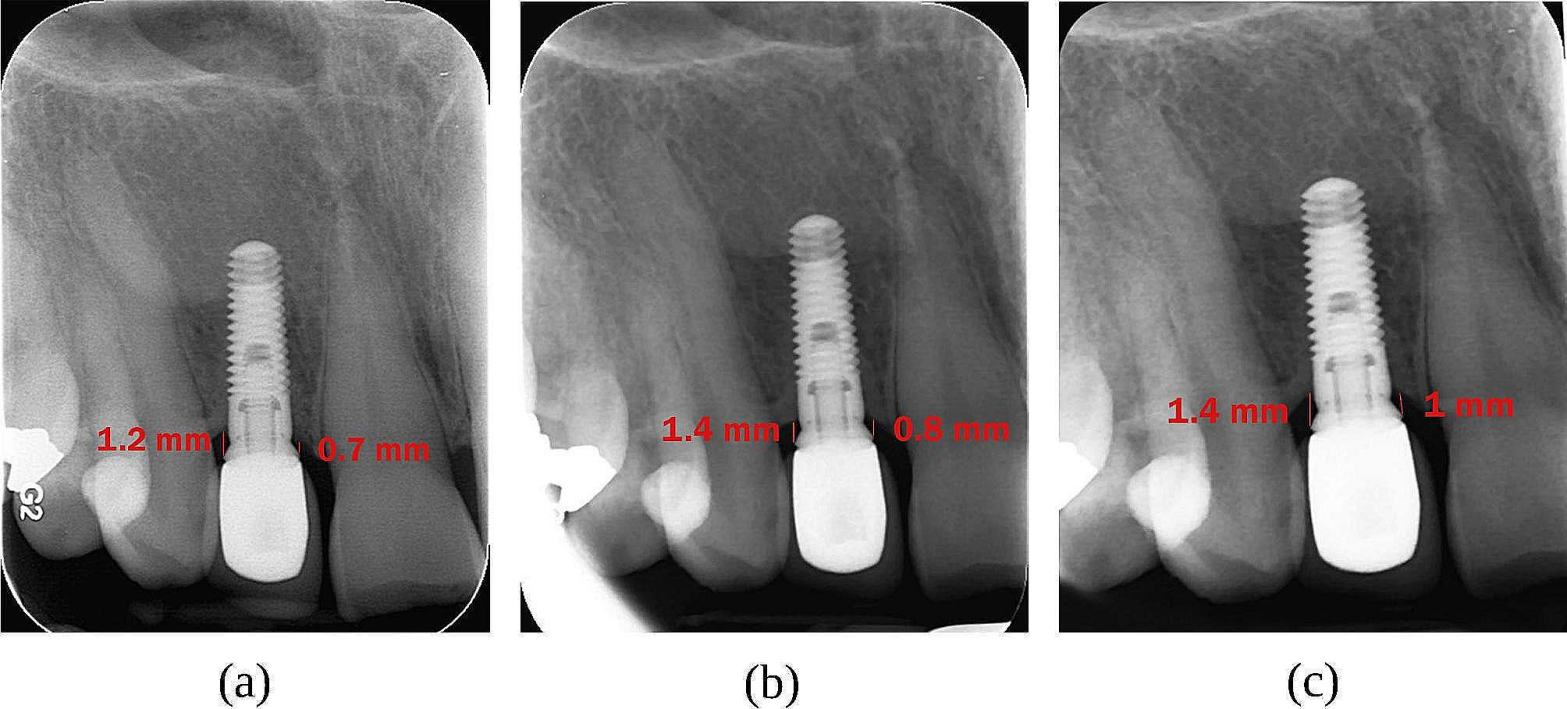
Radiographic assessments. (**a**) At the time of restoration delivery (baseline); (**b**) At six months follow-up; (**c**) At 12 months follow-up

The peri-implant marginal bone loss (MBL) was evaluated t_1_ = 0, t_2_ = 6, and t_3_ = 12 months after delivering the prostheses using a standardized periapical digital radiograph (Soredex Digora Optime, Finland) with a size-2 sensor and a film holder (Kerr, Kerr dental, Switzerland). All the follow-up and baseline radiographic images were standardized using the same film holder in parallel [38, 39]. An occlusal bite index made of high-viscosity additional silicone putty material (Zhermack SpA, Badia Polesine, Italy) was utilized for bite registration to guarantee that the film was placed correctly while taking follow-up radiographs, providing reproducible periapical radiographs [26, 32]. The same exposure settings were also applied in all the sessions for individual patients. According to Fig. 1, the distance between the abutment-restoration interface (crown margin) and the crestal bone-fixture interface was measured in Scanora software (SCANORA lite, Soredex, Finland) at the distal and mesial surfaces of the implant and time points t_1_, t_2,_ and t_3_. The diameter and length of the implants, which were already known, were utilized to calibrate the measurements in radiographs. The same clinician measured this distance three times for individual patients, and its mean level was reported to lower the error.

**Fig. 2:**
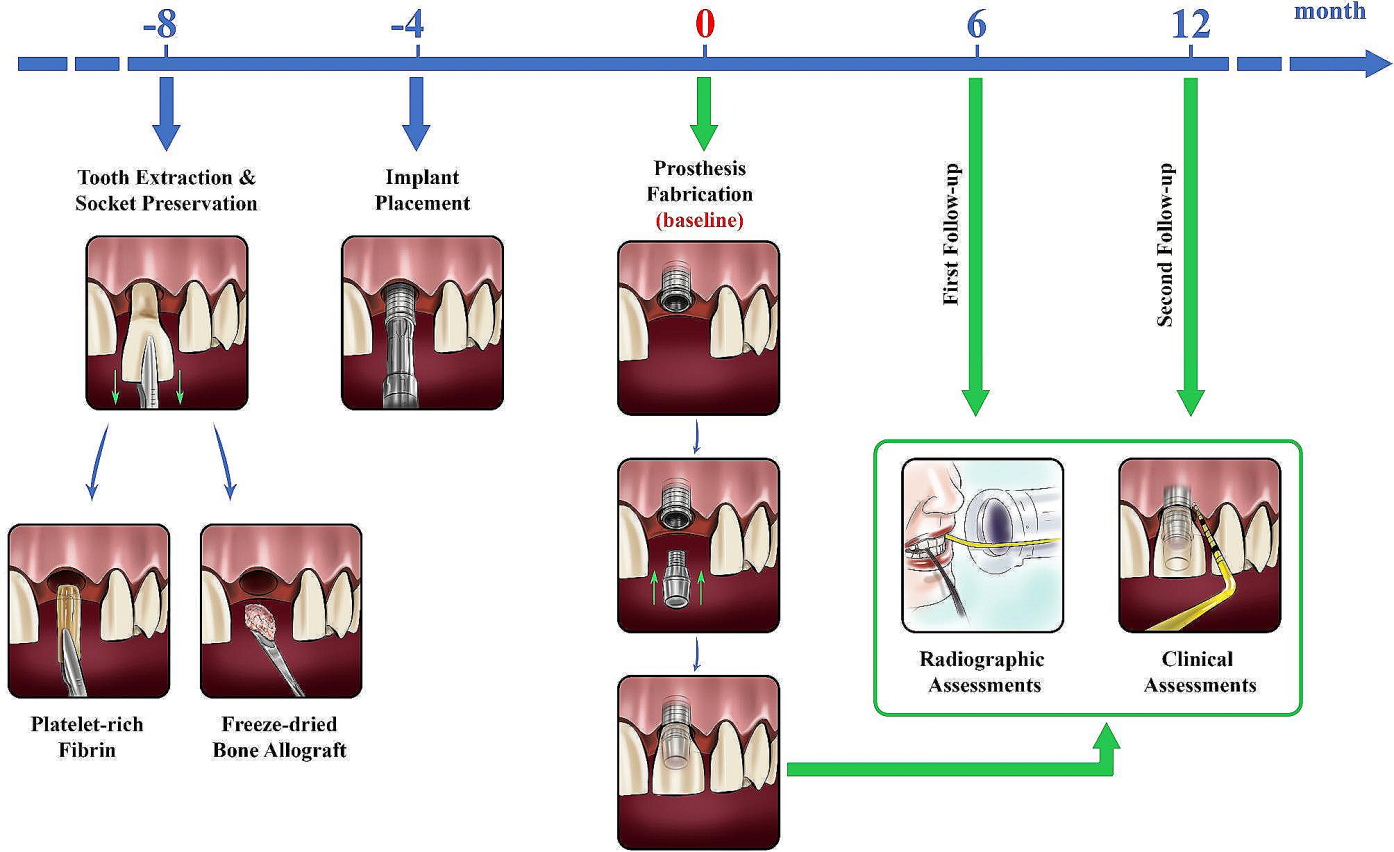
Time frame and intervention steps involved in structuring this experiment

A 0.2–0.25 N calibrated periodontal probe (Click-Probe, Kerr Corp, Orange, CA, USA) was employed to evaluate the soft tissue health by measuring bleeding on probing (BoP) at the distobuccal and mesiobuccal points and time points t_1_, t_2,_ and t_3_. The gingival recession (GR), defined as the difference in the mid-buccal distance from the free gingival margin to the restoration margin compared to the distance at baseline, was also measured as another periodontal indicator. An evaluator (H.Z.) blinded to the follow-up grouping performed the clinical and radiographic examinations. The steps involved in structuring this experiment are presented in Fig. 2.

### Statistical analysis

The Statistical Package for the Social Sciences (SPSS, Version 22.0) was used to analyze the data. The Generalized Estimating Equations (GEE) model with the Least Significant Difference (LSD) test as the pairwise comparison method was applied to compare the treatments regarding MBL during the study follow-up. Furthermore, the Binary logistic regression model and Cochran’s Q test were used to compare the risk of GR and BoP between the study groups and to test trends within the study groups. The level of significance was set at 0.05. The statistical studies were performed by a statistician blinded to the chosen treatment method, resulting in objective data analysis.

## Results

This randomized clinical trial recruited 40 patients referred to the Department of Periodontics at the Tehran University of Medical Sciences who needed tooth removal and potential implant treatment from September 2019 to June 2020. After reaching the anticipated sample number, the recruitment process ended. The patients were 37.5% (*n* = 15) males and 62.5% (*n* = 25) females. The average age of patients was 37.25 (22–50) and 30.81 (21–50) years in Groups 1 and 2, respectively. The characteristics of the cases in each study group are depicted in Table 1.

**Table 1:** Baseline characteristics of the study

Characteristics	Group 1 (FDBA)	Group 2 (PRF)
**Number of implants**	20	20
**Mean age (years)**	37.25 (22–50)	30.81 (21–50)
**Male/Female**	5/15	10/10
**Tooth extracted and replaced**: **Incisors/Premolars**	8/12	8/12
**Reason for tooth extraction**: **Inadequate crown-to-root ratio/extensive non-restorable caries/failed root canal treatment/ fractured roots**	6/6/5/3	5/6/5/4
**Implant location**: **Maxillary/Mandibular**	14/6	16/4
**Implant Length (mm)**: **8/10/11.5/13**	2/7/7/4	3/10/5/2
**Implant Diameter (mm)**: **3.3/3.7/4.1**	6/10/4	10/7/3

**Fig. 3:**
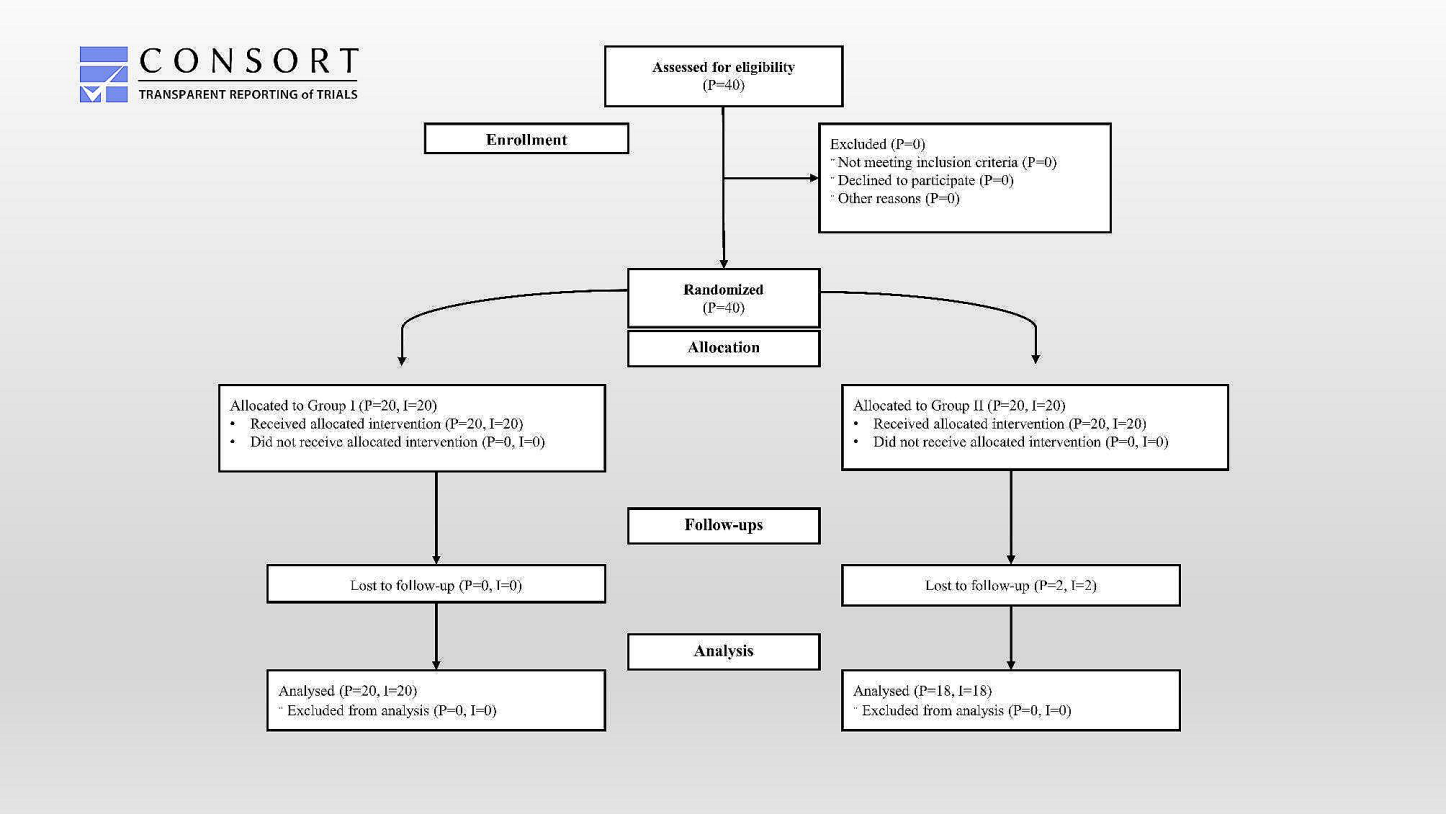
CONSORT flow chart of the included, excluded, and studied patients. (P = Number of Patients, I = Number of Dental Implants)

Due to inadequate cooperation in returning for the follow-up exams, two patients were excluded from the prosthetic stage (Fig. 3). Thirty-eight patients—23 females and 15 males—were ultimately evaluated for hard and soft tissue changes.

### Marginal bone loss around implants

The mean MBL around implants in the FDBA group were 0.19 ± 0.19 (mesial) and 0.29 ± 0.32 (distal) after six months and 0.36 ± 0.23 (mesial) and 0.40 ± 0.34 (distal) after 12 months. These measures were 0.15 ± 0.12 (mesial) and 0.07 ± 0.06 (distal) after six months, 0.26 ± 0.19 (mesial), and 0.20 ± 0.15 (distal) after 12 months in the PRF group.

**Fig. 4:**
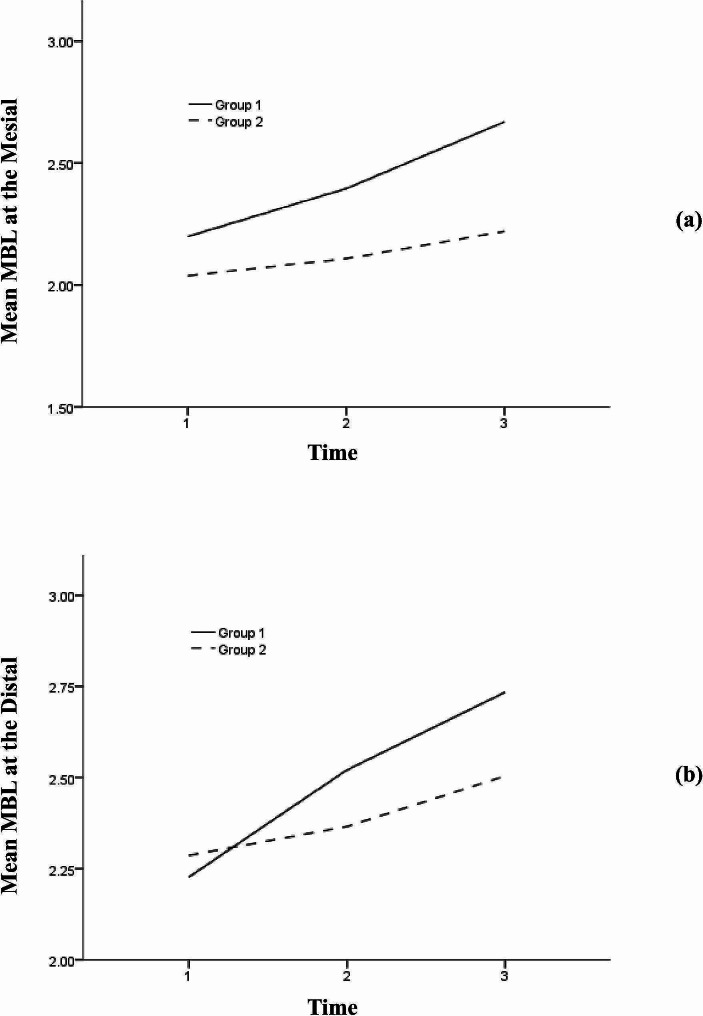
The trend of (**a**) Mesial MBL and (**b**) Distal MBL during the study period based on each group

The GEE model showed a significant interaction between group and time variables for all research outcomes. According to the between-group analysis, there was no significant difference in MBL between the study groups at either of the follow-up intervals (Table 2). The most considerable mean difference between the groups was seen at the second follow-up for the mesial MBL [MD = 0.448, 95% CI= (-0.284, 1.180)] (Fig. 4a). Furthermore, increasing trends were seen in both groups for both MBL responses (Fig. 4). Based on the within-group analysis, all pairwise comparisons were statistically significant (Table 3).

**Table 2:** Between-group comparison of MBL responses at each follow-up time

MBL Response	Time	Group 1 (FDBA)	Group 2 (PRF)	MD (95% CI)	*P*-value
Mesial	**Time 1**	2.2 ± 1.22	2.04 ± 1.12	0.1607 (-0.5636, 0.8850)	0.664
	**Time 2**	2.4 ± 1.35	2.11 ± 1.1	0.2867 (-0.4724, 1.0458)	0.459
	**Time 3**	2.67 ± 1.3	2.22 ± 1.06	0.4485 (-0.2839, 1.1809)	0.230
Distal	**Time 1**	2.23 ± 1.07	2.29 ± 1.36	-0.0597 (-0.8219, 0.7025)	0.878
	**Time 2**	2.52 ± 1.08	2.37 ± 1.38	0.1554 (-0.6188, 0.9297)	0.694
	**Time 3**	2.73 ± 1.06	2.5 ± 1.41	0.2301 (-0.5461, 1.0063)	0.561

Values were presented as mean ± standard deviation, MD: Mean difference (Group 1 – Group 2)

**Table 3:** Within-group comparison of MBL responses based on each group

MBL Response	Group	Comparison	MD (95% CI)	*P*-value
Mesial	**1**	**a**	-0.1960 (-0.2780, -0.1140)	< 0.001
		**b**	-0.4695 (-0.5698, -0.3692)	< 0.001
		**c**	-0.2735 (-0.3597, -0.1873)	< 0.001
	**2**	**a**	-0.0700 (-0.1317, -0.0083)	0.026
		**b**	-0.1817 (-0.2746, -0.0888)	< 0.001
		**c**	-0.1117 (-0.1718, -0.0515)	< 0.001
Distal	**1**	**a**	-0.2940 (-0.4335, -0.1545)	< 0.001
		**b**	-0.5070 (-0.6529, -0.3611)	< 0.001
		**c**	-0.2130 (-0.2718, -0.1542)	< 0.001
	**2**	**a**	-0.0789 (-0.1100, -0.0478)	< 0.001
		**b**	-0.2172 (-0.2895, -0.1449)	< 0.001
		**c**	-0.1383 (-0.1889, -0.0877)	< 0.001

MD: Mean difference a: Time 1 – Time 2 b: Time 1 – Time 3 c: Time 2 – Time 3

### Changes in GR and the frequency of positive BoP

In the FDBA group, 0 mm and 1 mm GR were observed in 9 (45%) and 11 (55%) subjects at six months post-delivery. These values were 16 (89%) and 2 (11%) for the PRF group. After 12 months, 9 (45%), 8 (40%), and 3 (15%) of subjects in Group 1 showed 0, 1, and 2 mm of gingival recession, respectively. Meanwhile, the PRF group’s results were similar to those at the six-month follow-up. According to the findings of the logistic regression model, the comparison of GR between the study groups was statistically significant at both follow-ups. That is, Group 2 decreased the risk of GR by almost 90% at both follow-ups.

The frequency of positive BoP around implants at six months was observed in four subjects of Group 1 [mesial: 1 (5.0%); distal: 3 (15%)] and ten subjects of Group 2 [mesial: 5 (27.8%); distal: 5 (27.8%)]. After 12 months, these values were positive in 10 Group 1 subjects [mesial: 4 (20.0%); distal: 6 (30.0%)] and 9 Group 2 subjects [mesial: 4 (22.2%); distal: 5 (27.8%)]. Although Group 2 seemed to have increased the risk of mesial BoP at both follow-ups and the risk of distal BoP at the second follow-up, the Risk Ratios (RRs) were not statistically significant (Table 4). Moreover, Cochran’s Q test revealed an increasing trend for both GR and BoP outcomes. However, that was not significant for GR in Group 2 (Table 5).

**Table 4:** Risk of GR and BoP in Group 2 compared to Group 1

Outcome	Category	Time	Group 1 (FDBA)	Group 2 (PRF)	RR (95% CI)	*P*-value
GR	0	2	9 (36)	16 (64)	0.102 (0.018, 0.568)	0.009
	1		11 (85)	2 (15)		
	0	3	9 (36)	16 (64)	0.113 (0.020, 0.631)	0.013
	1		10 (83)	2 (17)		
BoP (Mesial)	0	2	19 (59)	13 (41)	7.308 (0.763, 70.028)	0.085
	1		1 (17)	5 (83)		
	0	3	16 (53)	14 (47)	1.143 (0.240, 5.441)	0.867
	1		4 (50)	4 (50)		
BoP (Distal)	0	2	17 (57)	13 (43)	2.179 (0.439, 10.830)	0.341
	1		3 (38)	5 (63)		
	0	3	14 (52)	13 (48)	0.897 (0.220, 3.663)	0.880
	1		6 (55)	5 (45)		

Values were presented as number (percent), RR: Risk ratio, CI: Confidence interval

**Table 5:** Within-group comparison of GR and BoP

Outcome	Group	Comparison
GR	1	P12 < 0.001 P13 < 0.001
	2	NS
BoP (Mesial)	1	P13 = 0.043
	2	P12 = 0.019
BoP (Distal)	1	P13 = 0.008
	2	P12 = 0.043 P13 = 0.043

NS: Not Significant; P12: Comparison of time 1 and time 2; P13: Comparison of time 1 and time 3

## Discussion

The principles of guided bone regeneration, minimally invasive tooth extraction, protection of the initial blood coagulum, and introduction of biomaterials within the extraction socket are all part of the ARP protocol [40], aimed at preserving the alveolar bone’s initial volume and structural integrity to achieve an aesthetically pleasing and functionally sound prosthetic reconstruction following implant therapy [41]. PRF, a complex natural scaffold derived from centrifuged blood and purely autologous, is one such scaffold used in soft and hard tissue regeneration [42]. Numerous studies showing osteoblast and gingival fibroblast proliferation and differentiation have demonstrated the applicability of PRF as a biologically active scaffold [26, 43, 44]. On the other hand, allogenic bone graft materials, such as mineralized FDBA, are among the most widely used biomaterials in alveolar ridge preservation [16].

This study compared the hard tissue stability and soft tissue changes around dental implants placed in the preserved extraction sites with FDBA or PRF by comparing MBL, BoP, and gingival recession six and 12 months after loading the implants. At 6 and 12 months, the FDBA and PRF groups’ mean MBL at the mesial or distal of implants did not vary significantly from one another. Compared to the FDBA group, the mean gingival recession was considerably lower in the PRF group. The gingival level was stable in 89% of the implants placed in the preserved bone by PRF, while it receded 1–2 mm in 55% of cases in the FDBA group; however, BoP was not significantly different in both groups. Therefore, the null hypothesis was partially rejected.

Galindo- Moreno et al. defined a cut-off value of 0.44 mm MBL six months post-loading to ensure a high implant success rate after one year [10]. The initial bone loss after implant loading in the present study was less than 0.29 mm at six months, which might indicate a high long-term success rate in both groups. Similarly, this study showed that FDBA and PRF resulted in high soft and hard tissue stability after loading the implants placed in preserved post-extraction sites. At the same time, there was no need for further bone grafting at implant placement, which was in line with other studies [31, 45] that justified that the need for further bone grafting at implant placement was reduced using a ridge preservation procedure. To minimize bias in assessing the result of this study, the diameter of the implants was selected so that at least 1 mm of buccal bone was maintained after implant insertion. Hence, the implants were narrow or standard in diameter according to the existing bone.

Whether PRF for bone regeneration is more advantageous than bone grafting alone is still disputed. A systematic review by Castro et al. assessed the efficacy of L-PRF in preserving the alveolar ridge for implant placement. They concluded that applying L-PRF is easy, cost-beneficial, and has optimal biological properties [46]. As supported by several other studies [25, 47], this procedure minimizes morbidity and pain, speeds up wound healing, and new soft/hard tissue regeneration [48, 49]—all of which contribute to enhanced long-term implant stability by minimizing marginal and buccal bone loss [26, 50, 51]. Furthermore, certain systematic reviews have demonstrated encouraging results for PRF’s potential to maintain bone volume after ARP interventions, either by itself or in combination with other grafting materials [52, 53].

A randomized controlled clinical trial with human histologic samples showed that using L-PRF, produced by the same protocol as this study, in extraction sockets could reduce horizontal and vertical bone loss and enhance new bone formation in histomorphometric analysis after three months [54]. In this study, PRF, without using any additional biomaterial as a space maintainer, yielded comparable results at follow-ups with FDBA. In a similar study by Azangookhiavi et al., after three months following implant insertion, PRF application in extraction sockets produced identical ridge width and height outcomes to FDBA [16]. The current research demonstrated that the height of preserved peri-implant bone following ARP could be maintained one year after implant loading in these two groups. Clark et al. [35] also examined the effectiveness of A-PRF alone or in combination with FDBA in enhancing alveolar dimensional stability and vital bone formation in ARP. Their results showed no significant differences between study groups regarding alveolar width resorption; compared to the FDBA group, the A-PRF group had a significantly more vital bone. They have credited the concentrated and intrinsic growth factors found in A-PRF for the increased formation of vital bone.

Hehn et al. compared the application of PRF in partial thickness soft tissue flaps around dental implants. They failed to show the advantage of using PRF with the split-flap technique in thickening an initially thin mucosa around implants [55]. However, in the present study, flap elevation or splitting, which may result in nutritional deficiencies of the site, was not performed, and the results of PRF indicated minor soft tissue changes and less gingival recession compared to FDBA at six and 12 months. In the present study, gingival recession increased to a small extent over 12 months after restoration delivery only in the FDBA group. In general, our findings revealed the superiority of PRF to FDBA regarding soft tissue health and recession. It can be assumed that using PRF for ARP could help form a higher-quality soft tissue more resistant to recession. This finding can be of great importance for using PRF in the esthetic zone where gingival recession can jeopardize the success rate of the treatment. PRF membranes are determined to be a predictable choice in treating gingival recessions and are even regarded as an alternative to gingival grafts due to their regenerative potential [56].

To avoid group heterogeneity, only simple sockets without any buccal bone defect were selected in the present study. Furthermore, the teeth with a lack of keratinized gingiva were excluded since one of the essential factors influencing the marginal bone loss and gingival recession after implant loading is the vertical mucosal thickness; a thin mucosa (≤ 2 mm) can be significantly associated with more complications [57]. Furthermore, other studies have also used the resorbable collagen sponge similarly utilized in this study to stabilize the socket wound in the FDBA group [35, 36]. Any gain in new bone formation from utilizing this dressing could be ascribed to the graft material itself [35]. There are also disadvantages to using PRF: it requires a centrifuge for preparation, and blood should be taken from the patients, which is invasive. Also, PRF should be prepared right after blood collection and applied immediately in the extraction socket.

The present study also had certain limitations; it would be ideal to study only one region within the jaw for ARP and implant placement. The number of teeth in each area was limited in our study. Thus, we could not assess the soft and hard tissue indices separately for different sites, and the samples could not be standardized in this respect. Similar studies with larger sample sizes and histological analysis on samples obtained at implant site preparation are required to obtain more accurate results. Also, the synergistic effect of PRF in combination with other materials should be evaluated in long-term future studies. The other dimensions of the preserved ridge around implants, like the width of crestal bone, the palatal and buccal bone plate thickness, and bone quality, as well as other peri-implant diseases indicators, such as post-loading probing depth, should be evaluated in long-term future studies.

## Conclusions

Considering all limitations, this study highlighted the comparable efficacy of PRF to FDBA for marginal bone and alveolar ridge preservation following implant loading. There was no significant difference between PRF and FDBA regarding bleeding on probing outcomes; however, PRF outperformed FDBA in minimizing gingival recession; therefore, considering its optimal efficacy, easy preparation, and low cost, it could be a promising substitute material for ridge preservation.

## Data Availability

The data supporting this study’s findings are available from the corresponding author upon reasonable request.

## Abbreviations

PRF: Platelet-rich Fibrin
FDBA: Freeze-dried Bone Allograft
ARP: Alveolar Ridge Preservation
BoP: Bleeding on Probing
MBL: Marginal Bone Loss
CBCT: Cone-Beam Computed Tomography
Ncm: Newton centimeters
GR: Gingival Recession
GEE: Generalized Estimating Equations
LSD: Least Significant Difference
RRs: Risk Ratios
